# Berberine in oral diseases: molecular mechanisms and therapeutic implications

**DOI:** 10.3389/fphar.2026.1853738

**Published:** 2026-07-02

**Authors:** Siyuan Wu, Bowen Wang, Jiaqi Zhu, Hongli Chen, Yuankun Zhai

**Affiliations:** 1 School of Stomatology, Henan University, Kaifeng, China; 2 Kaifeng Key Laboratory of Periodontal Tissue Engineering, Kaifeng, China; 3 Department of Stomatology, Henan Provincial People’s Hospital, Zhengzhou, China

**Keywords:** berberine, dental caries, endodontic diseases, oral cancer, oral mucosal diseases, periodontitis

## Abstract

Oral diseases, including periodontitis, oral mucosal diseases, dental caries, endodontic diseases, and oral cancer, are among the most prevalent diseases worldwide and impose substantial health and economic burdens. Current therapeutic strategies are often limited by incomplete efficacy, drug resistance, or adverse effects, highlighting the need for alternative pharmacological approaches. Berberine, a natural isoquinoline alkaloid derived from medicinal plants such as *Coptis chinensis* and *Phellodendron* species, has attracted increasing attention because of its broad pharmacological properties. Accumulating evidence indicates that berberine exerts antimicrobial, anti-inflammatory, antioxidant, bone-regulating, and antitumor effects, which are closely associated with key pathogenic mechanisms involved in oral diseases. This review systematically summarizes the pleiotropic pharmacological effects of berberine and discusses its mechanistic roles in the management of periodontitis, recurrent aphthous ulcers, oral candidiasis, dental caries, pulpitis and apical periodontitis, and oral cancer. In addition, recent advances aimed at improving the bioavailability of berberine through novel drug delivery systems, including nanocarriers and hydrogels, are summarized. Finally, current challenges and future research directions are proposed to facilitate the clinical translation of berberine-based therapies in oral diseases.

## Graphical Abstract

**Figure:**
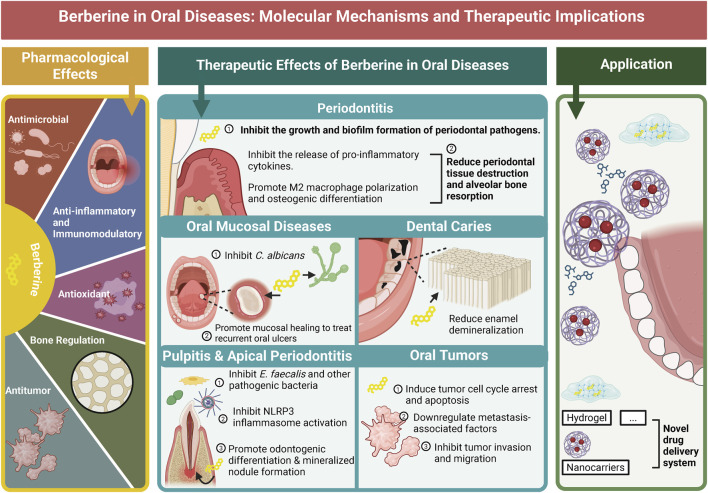

## 1 Introduction

Oral diseases rank among the most prevalent diseases worldwide, imposing substantial health and economic burdens and profoundly undermining patients’ quality of life (Peres et al., 2019). It is estimated that more than 3.5 billion people globally are affected by oral diseases, making them a major global public health issue (Watt et al., 2019). Oral diseases comprise a wide spectrum of conditions, including periodontitis, oral mucosal diseases, dental caries, endodontic diseases and oral tumors. Periodontitis is a chronic, multifactorial inflammatory disease associated with complex interactions between the dental plaque biofilm and the host immune response, with approximately 11% of the global population suffering from severe periodontitis. As the disease progresses, periodontitis leads to irreversible destruction of periodontal supporting tissues, such as the alveolar bone and periodontal ligament, ultimately resulting in tooth loss. In addition, periodontitis may also contribute to the development of certain systemic chronic diseases (Kwon et al., 2021). Among oral mucosal diseases, recurrent aphthous ulcers are characterized by periodicity and recurrence, and the burning pain during episodes is often unbearable, severely affecting patients’ quality of life. And oral candidiasis is an opportunistic infectious oral mucosal disease predominantly caused by *Candida albicans* (*C. albicans*) and commonly occurs in conditions such as immunodeficiency, xerostomia, denture wearing, or long-term antibiotic use. At present, conventional pharmacological treatments for recurrent aphthous ulcers and oral candidiasis have certain limitations (He et al., 2021; Gharibpour et al., 2021). Similar to periodontitis, dental caries is also one of the most prevalent oral diseases and a leading cause of tooth loss, affecting large populations worldwide. Although the global incidence of dental caries has declined in recent years, its widespread occurrence across all age groups remains an unresolved problem (Frencken et al., 2017). Endodontic diseases primarily encompass pulpitis and apical periodontitis, which are most commonly triggered by microbial invasion of the dental pulp and root canal system. Without timely intervention, pulpal inflammation may progress to pulpal necrosis and subsequent periapical inflammation, ultimately leading to periapical tissue destruction and tooth loss (Duncan et al., 2023). Cancers of lip and oral cavity rank as the 16th most common cancers worldwide and represent a significant global health concern, with a continuously increasing incidence in recent years. However, in developing countries, public awareness of their pathogenesis and risk factors remains generally inadequate (Kijowska et al., 2024). Furthermore, with advances in research in recent years, a growing body of evidence has demonstrated that oral diseases may also be associated with systemic conditions such as cancer, cardiovascular diseases, and neurodegenerative disorders (Botelho et al., 2022). Therefore, the prevention and timely treatment of oral diseases are of paramount importance.

Berberine is a yellow crystalline quaternary isoquinoline alkaloid that can be extracted from the roots, stems, rhizomes, and bark of several medicinal plants, including *Coptis chinensis* Franch [Ranunculaceae], *Berberis vulgaris* L [Berberidaceae], and *Phellodendron amurense* Rupr [Rutaceae] (Neag et al., 2018). Its molecular formula is C_20_H_18_NO_4_^+^, with a molecular weight of 336.36 g/mol. The extraction and use of berberine from botanical sources have been practiced for centuries in traditional Chinese medicine, where it has been commonly used to manage gastrointestinal disorders and infections (Xu et al., 2025). In modern medicine, berberine has been demonstrated to exert a broad range of pharmacological effects, including antimicrobial, anti-inflammatory, antioxidant, bone-modulatory, and antitumor activities (Rui et al., 2020; An et al., 2022; Coppinger et al., 2024). These pleiotropic properties provide therapeutic perspectives for the management of oral diseases.

Compared with prior reviews that mainly addressed the overall health-related effects of berberine, including its anti-inflammatory, antioxidant, immunomodulatory, and anticancer activities (Almatroodi et al., 2022; Hsu et al., 2024; Li Z. et al., 2023; Shakeri et al., 2024; Shi et al., 2025; Hashim et al., 2025; Mohammadian Haftcheshmeh and Momtazi-Borojeni, 2021), this review focuses specifically on the role of berberine in oral diseases. It summarizes the evidence related to periodontitis, dental caries, oral candidiasis, endodontic diseases, and oral cancer, while further discussing the physicochemical and pharmacokinetic properties, pharmacological limitations, safety concerns, toxicity, and potential drug interactions of berberine. Although berberine-based drug delivery systems and nanoformulations have been discussed in cancer-related contexts, their disease-specific relevance and intraoral delivery advantages in oral medicine remain insufficiently summarized (Almatroodi et al., 2022; Hsu et al., 2024). Therefore, we highlight berberine-based nanoformulations for intraoral application, which may help overcome the poor solubility, limited bioavailability, rapid clearance, and insufficient local retention of free berberine. These delivery systems may provide advantages such as controlled release, improved mucosal or periodontal retention, enhanced penetration into oral biofilms and inflamed tissues, and reduced systemic exposure. By linking disease-specific mechanisms with pharmacological constraints and delivery strategies, this review aims to provide a balanced and evidence-oriented assessment of the current experimental and clinical evidence for berberine-related interventions in oral medicine, while emphasizing the need for further well-designed clinical studies.

## 2 Methodology of literature search and selection

This review was designed as a narrative review with an integrative approach. Relevant literature was searched in PubMed, Web of Science, and Google Scholar for publications from January 1, 2004, to January 1, 2026. The main search strategy using Boolean operators was as follows: “berberine” AND (“oral disease” OR “periodontitis” OR “dental caries” OR “oral candidiasis” OR “recurrent aphthous stomatitis” OR “endodontic disease” OR “pulpitis” OR “apical periodontitis” OR “oral cancer”). Eligible studies included *in vitro* experiments, animal studies, clinical studies, and relevant reviews. Studies involving pure berberine or its salts, berberine-containing extracts, and berberine-based complex formulations were considered. Studies were excluded based on the following criteria: non-English publication, absence of berberine or berberine-containing preparations, and lack of relevance to oral diseases. Conference abstracts, editorials, letters, and studies with insufficient methodological or experimental information were also excluded.

## 3 Pharmacological effects of berberine

### 3.1 Antimicrobial effects of berberine

**FIGURE 1:**
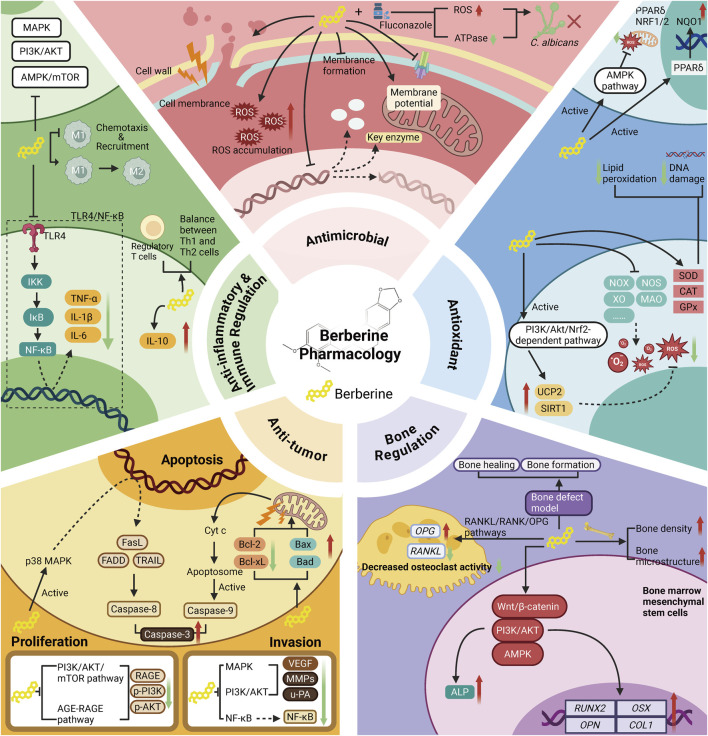
Pharmacological Effects of Berberine. Antimicrobial activity, anti-inflammatory and immune regulation effects, antioxidant effects, anti-tumor properties, and bone regulation effects. Fas-associated death domain protein (FADD), Fas ligand (FasL), Reactive oxygen species (ROS), TNF-related apoptosis-inducing ligand (TRAIL), Matrix metalloproteinases (MMPs), Vascular endothelial growth factor (VEGF), Urokinase-type plasminogen activator (u-PA), Nuclear factor-κB (NF-κB), *Candida albicans* (*C. albicans*), Peroxisome proliferator–activated receptor delta (PPARδ), Superoxide dismutase (SOD), Catalase (CAT), Glutathione peroxidase (GPx), NADPH oxidase (NOX), Xanthine oxidase (XO), Nitric oxide synthase (NOS), Monoamine oxidase (MAO), Uncoupling protein 2 (UCP2), Sirtuin 1 (SIRT1), Alkaline phosphatase (ALP), and AMP-activated protein kinase (AMPK). Created in BioRender.

Berberine exerts antimicrobial activity through multiple mechanisms (Figure 1). Evidence indicates that berberine can disrupt the bacterial cell wall, leading to cytoplasmic condensation and leakage of intracellular contents, while concurrently inhibiting DNA and protein synthesis, which blocks microbial division and growth and ultimately causing bacterial death. These effects appear to be both concentration- and time-dependent (Kang et al., 2015). Berberine may also interfere with carbohydrate metabolism and promote the accumulation of reactive oxygen species (ROS), resulting in oxidative damage and impaired synthesis of DNA, proteins, and lipids, which ultimately triggers cell death (Du et al., 2020). In addition, co-administration of berberine with fluconazole has been reported to increase mitochondrial membrane potential, reduce intracellular ATP levels, inhibit ATP synthase activity, and elevate endogenous ROS, producing synergistic antimicrobial effects against fluconazole-resistant *C. albicans* (Xu et al., 2009). Moreover, berberine can alter the composition of the microbial cell membrane and inhibit efflux pump activity, imposing osmotic stress and activating oxidative stress responses that contribute to its antimicrobial efficacy (Gupta et al., 2023). Beyond these mechanisms, berberine has also been proposed to inhibit biofilm formation, reduce microbial adhesion and invasiveness, decrease lipopolysaccharide (LPS) levels, and competitively inhibit the key coenzyme pyridoxal 5′-phosphate (PLP), suppressing the activity of PLP-dependent enzymes (e.g., tryptophanase), disrupting microbial metabolism, and reducing microbial adaptability to environment stress (Zhou W. et al., 2025).

### 3.2 Anti-inflammatory and immunomodulatory effects of berberine

Berberine exerts anti-inflammatory and immunomodulatory effects by regulating multiple inflammation-related molecular mechanisms and by modulating diverse immune cell populations and their functional states (Figure 1). Mechanistically, berberine targets key intracellular signaling pathways, including Toll-like receptor 4/nuclear factor-κB (TLR4/NF-κB), mitogen-activated protein kinase (MAPK; e.g., p38 MAPK and extracellular signal-regulated kinase 1/2 [ERK1/2]), phosphoinositide 3-kinase/protein kinase B (PI3K/AKT), and AMP-activated protein kinase/mammalian target of rapamycin (AMPK/mTOR), influencing immune-cell activation, cytokine production, and the overall inflammatory response. For example, by inhibiting IκB kinase β (IKKβ) phosphorylation and NF-κB activation, berberine decreases the transcription of proinflammatory cytokines such as TNF-α, IL-1β, and IL-6, which are central regulators of inflammatory progression (Zhou W. et al., 2025; Feng et al., 2025). In addition to suppressing proinflammatory mediators, berberine can increase the production of anti-inflammatory cytokines (e.g., IL-10), consistent with its immunosuppressive and anti-inflammatory effects (Yang et al., 2024). Berberine also inhibits inflammasome activation and attenuates oxidative stress, which may act in concert to limit tissue injury (Habiba et al., 2025). A growing body of both *in vitro* and *in vivo* evidence indicates that berberine significantly inhibits inflammatory biomarkers, oxidative stress markers, and apoptosis in experimental models of colitis, arthritis, and neuroinflammation (Jia et al., 2020; Kodi et al., 2024; Yuan et al., 2025). In macrophages, berberine limits secretion of inflammatory cytokines (e.g., TNF-α) and chemokines (e.g., CCL2, CCL4, and CCL5) by suppressing chemotaxis and recruitment of proinflammatory M1 macrophages while promoting activation of anti-inflammatory M2 macrophages. This macrophage polarization is critical for controlling chronic inflammation and limiting tissue damage (Noh et al., 2022). Moreover, berberine contributes to immune homeostasis by modulating the Th1/Th2 balance, promoting regulatory T-cell generation, and restraining excessive immune activation (Yang et al., 2024; Yang et al., 2022). Notably, inhibition of NF-κB signaling by berberine not only reduces inflammatory mediator production but also protects tissues from immune-mediated injury, as demonstrated in models of autoimmune hepatitis and obesity-associated inflammation (Yang et al., 2024; Noh et al., 2022). Moreover, these immunoregulatory effects may confer antiviral activity. Berberine has been reported to enhance antiviral responses against viruses such as SARS-CoV-2 by promoting IFN-γ production in CD8^+^ T cells and inhibiting histamine release from mast cells (Wang Z. Z. et al., 2021). Overall, the immunomodulatory properties of berberine may contribute not only to suppression of pathogenic microorganisms but also to maintenance of balanced host immune responses, supporting its potential as a versatile supplementary treatment for oral inflammatory diseases.

### 3.3 Antioxidant effects of berberine

Berberine also exerts antioxidant effects through multiple mechanisms (Figure 1). Experimental evidence indicates that berberine can directly scavenge free radicals and markedly upregulate the activity of endogenous antioxidant enzymes, including superoxide dismutase (SOD), catalase (CAT), and glutathione peroxidase (GPx), reducing lipid peroxidation and DNA damage (Shakeri et al., 2024). In addition, berberine has been reported to inhibit several oxidant-producing enzymes, such as NADPH oxidase (NOX), xanthine oxidase (XO), nitric oxide synthase (NOS), and monoamine oxidase (MAO), leading to decreased generation of free radicals and ROS and attenuation of oxidative stress. Other studies suggest that berberine activates the PI3K/Akt/Nrf2-dependent pathway to counteract oxidative stress and increases the expression of uncoupling protein 2 (UCP2) and sirtuin 1 (SIRT1), limiting ROS production and preventing mitochondrial oxidative injury (Purwaningsih et al., 2023). Activation of AMPK signaling by berberine, which reduces mitochondrial ROS generation and improves cellular energy metabolism, has also been proposed as an important mechanism underlying its antioxidant actions (García-Muñoz et al., 2024). In studies of ischemic stroke, berberine was further shown to directly activate peroxisome proliferator–activated receptor delta (PPARδ) and initiate transcriptional regulation, promoting the expression of PPARδ, nuclear factor erythroid 2–related factor 1/2 (NRF1/2), and NAD(P)H quinone oxidoreductase 1 (NQO1), which collectively enhance ROS clearance and suppress oxidative stress (Sun et al., 2018).

### 3.4 Bone regulation effects of berberine

Berberine has the potential to treat skeletal disorders, including osteoporosis (Chen et al., 2021; Eid et al., 2025), calvarial defects (Wang et al., 2021a), and periodontitis (Mi et al., 2024), by inhibiting bone resorption and promoting bone formation (Figure 1). Evidence indicates that berberine significantly enhances the proliferation of osteogenic cells (e.g., osteoblasts) and augments their differentiation toward a mature osteoblastic phenotype. Additionally, it reduces osteoclast number and activity, suppressing osteoclastic bone resorption (Cui et al., 2023). Mechanistically, berberine promotes osteogenic differentiation of bone marrow–derived mesenchymal stem cells by activating Wnt/β-catenin, PI3K/AKT, and AMPK signaling, upregulating osteogenesis-related genes such as *RUNX2*, *OSX*, *OPN*, and *COL1*, and increasing alkaline phosphatase (ALP) activity (Qin et al., 2024). In parallel, berberine modulates the RANKL/RANK/OPG axis by increasing *OPG* expression and decreasing *RANKL* levels, inhibiting osteoclast activity and improving the resorptive balance. In an experimental calvarial defect model, berberine delivered through a porous calcium phosphate scaffold significantly promoted bone healing and increased new bone formation (Wang et al., 2021a). Consistent with these findings, animal studies have shown that berberine increases bone mineral density and improves bone microarchitecture, supporting its therapeutic potential for osteoporosis (Chen et al., 2021; Eid et al., 2025).

### 3.5 Antitumor properties of berberine

Berberine exerts antitumor effects primarily by inhibiting tumor cell proliferation, invasion, and migration, inducing cell-cycle arrest, and promoting autophagy and apoptosis (Liu et al., 2019) (Figure 1). Berberine has been reported to suppress the proliferation of multiple cancer cell lines, including A549 lung cancer cells (Mistry et al., 2017), SKOV3 ovarian cancer cells (Su et al., 2015), HCT-15 colorectal adenocarcinoma cells (Agnarelli et al., 2018), CaSki cervical cancer cells (Kalaiarasi et al., 2016), and BIU-87 and T24 bladder cancer cells (Lu et al., 2015). Mechanistically, berberine downregulates the expression of cell cycle–related proteins, particularly by modulating cyclin-dependent kinase (CDK) activity, resulting in cell-cycle arrest at the G1 phase or the G2/M phase (Ling et al., 2023). In addition, berberine can activate both extrinsic and intrinsic apoptotic pathways, converging on caspase-3 activation to initiate the apoptotic cascade and induce tumor cell death. Moreover, by inhibiting signaling pathways such as MAPK, PI3K/AKT, and NF-κB, berberine downregulates the expression of key pro-tumorigenic factors, including vascular endothelial growth factor (VEGF), matrix metalloproteinase (MMP)-2, MMP-9, and urokinase-type plasminogen activator (u-PA), reducing endothelial cell proliferation and extracellular matrix (ECM) degradation and ultimately suppressing tumor cell invasion and migration (Lopez and Tait, 2015).

## 4 Therapeutic applications of berberine in oral diseases

### 4.1 The application of berberine in Treatment of Periodontitis

**FIGURE 2:**
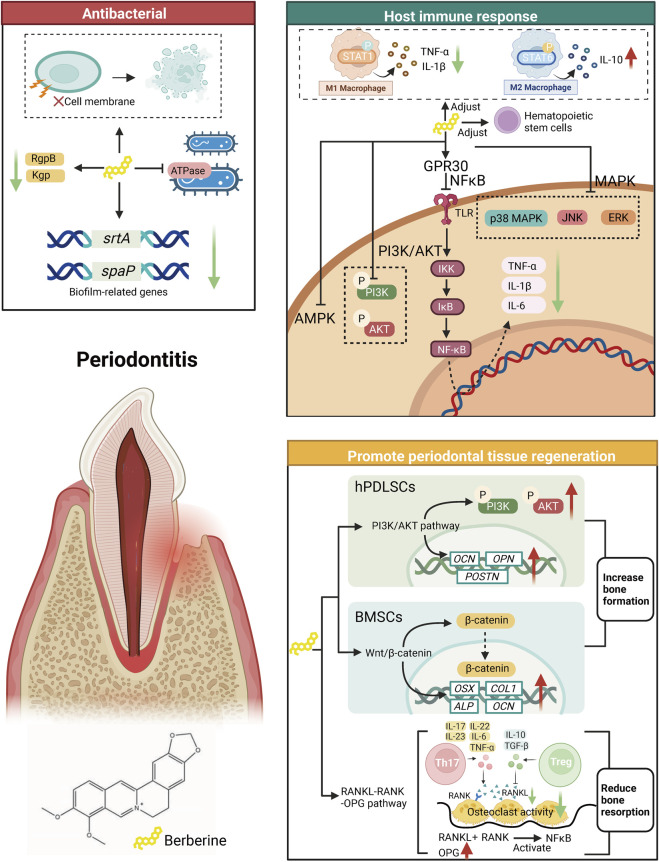
Mechanisms of Berberine in the Treatment of Periodontitis. Inhibition of periodontal pathogens, modulation of the host immune response, and tissue repair and periodontal regeneration. *Sortase A* (*srtA*), Human periodontal ligament stem cells (hPDLSCs), and Bone marrow–derived mesenchymal stem cells (BMSCs). Created in BioRender.

Oral microbial dysbiosis disrupts microbial homeostasis and elicits destructive host immune-inflammatory responses, ultimately leading to progressive breakdown of periodontal supporting tissues and alveolar bone resorption. The initiation and progression of periodontitis closely associate with key periodontal pathogens, including *Porphyromonas gingivalis* (*P. gingivalis*), *Fusobacterium nucleatum* (*F. nucleatum*), and *Fusobacterium necrophorum* (*F. necrophorum*). Berberine has antibacterial and anti-inflammatory properties and can suppress bone resorption while promoting bone formation. This highlights its potential as an adjunct therapy for periodontitis management (Figure 2).

#### 4.1.1 Berberine inhibits the periodontal pathogens and disrupts the initiating events in periodontitis

The overgrowth of periodontal pathogens and biofilm formation constitute central initiating events in periodontitis. Berberine chloride and *Phellodendron* bark extract (PBE) reduce the relative abundance of key periodontal pathogens such as *P. gingivalis* within the oral microbiome and, in both planktonic cultures and complex biofilm models, effectively promote the re-establishment of microbial homeostasis, an effect that is further enhanced through synergy with commensal genera such as *Streptococcus*, *Neisseria*, and *Haemophilus* (Inokuchi et al., 2025; Okuda et al., 2023). Through these complementary actions—attenuating pathogen proliferation, downregulating virulence factor expression, and inhibiting biofilm formation—berberine provides a biologically plausible infection-control foundation for the management of periodontitis.

Berberine inhibits the proliferation of periodontal pathogens and attenuates the release of virulence factors. *In vitro*, Zhang et al. demonstrated that berberine exerted potent growth-inhibitory effects against *P. gingivalis*, with a minimum inhibitory concentration (MIC) of 31.3 μg/mL, and dose-dependently suppressed the activity of the secreted gingipain RgpA, reducing its ability to damage periodontal tissues (Zhang et al., 2019). In addition, a poly (lactic-co-glycolic acid) (PLGA) microsphere gel loaded with berberine, developed by Mi et al., significantly inhibited the growth of *F. necrophorum in vitro* (Mi et al., 2024). Berberine has been reported to disrupt the phospholipid bilayer of the bacterial membrane, increasing membrane permeability and causing leakage of intracellular contents, ultimately leading to bacterial death. Moreover, berberine can selectively inhibit bacterial ATPase activity, impair ATP synthesis, and induce energy depletion, preventing the maintenance of essential survival processes such as biofilm formation and virulence factor secretion and further enhancing pathogen clearance (Li et al., 2017; Zhao et al., 2023). Li et al. constructed bismuth-doped carbon dots functionalized with structure modified berberine (BiCD-Ber) and found that this platform could eradicate *P. gingivalis* across multiple bacterial states, including planktonic bacteria, biofilm-associated bacteria, and intracellular bacteria—phenotypes that contribute to immune evasion, antimicrobial tolerance, and persistence. Notably, BiCD-Ber blocked the catalytic sites of *P. gingivalis* gingipains via bismuth ions, while Ber-NH_2_ acted synergistically with BiCD to enhance inhibition of gingipains (RgpB and Kgp), limiting the invasive potential of *P. gingivalis* toward host tissues (Li X. et al., 2025).

Berberine can also inhibit subgingival biofilm formation and impede bacterial colonization. Periodontal pathogens predominantly reside within biofilms in periodontal pockets, a lifestyle that markedly enhances antimicrobial tolerance and pathogenicity. By downregulating biofilm-associated gene expression and disrupting biofilm structural integrity, berberine limits pathogenic adhesion and establishment. Li et al. developed an engineered probiotic (Bif@BIP) capable of targeted delivery of berberine to hypoxic regions of the periodontal pocket. Upon near-infrared irradiation, berberine was released and inhibited *P. gingivalis* serine acetyltransferase activity, perturbing sulfur metabolism, weakening resistance to oxidative stress, and ultimately suppressing *P. gingivalis* biofilm formation (Li S. et al., 2025). Zhou et al. designed a nano/micro hydrogel microsphere (PDA/berberine@Gel@BMP9-PDLSC) that provides sustained berberine release and, in a rat model of periodontitis with persistent *F. nucleatum* infection, significantly reduced *F. nucleatum* biofilm thickness and the proportion of viable bacteria (Zhou S. et al., 2025). In addition, Tu et al. reported *in vitro* that berberine dose-dependently inhibited biofilm formation in a co-culture system of human gingival fibroblasts and U937 macrophages stimulated with *P. gingivalis* LPS, accompanied by downregulation of biofilm-related genes such as *srtA* and *spaP* (Tu et al., 2013).

#### 4.1.2 Berberine modulates the host immune response to attenuate inflammation

The initiation and progression of periodontitis can largely be attributed to dysregulated host immune responses, characterized by excessive production of proinflammatory mediators, dysregulated immune-cell activation, and sustained activation of inflammatory signaling pathways. Emerging evidence suggests that berberine ameliorates periodontitis through modulation of multiple signaling cascades, including NF-κB, MAPK, PI3K/AKT, and AMPK pathway involved in inflammation, oxidative stress, metabolism, and cell survival (Zhou W. et al., 2025).

Berberine modulates immune-cell polarization to rebalance the inflammatory microenvironment. Dysregulated macrophage M1/M2 polarization represents a key pathogenic event in periodontitis: M1 macrophages secrete high levels of proinflammatory cytokines, including TNF-α and IL-1β, exacerbating tissue injury, whereas M2 macrophages produce anti-inflammatory mediators such as IL-10, suppress inflammation, and support tissue repair. In a mouse model of type 2 diabetes mellitus (T2DM)–associated periodontitis, Xia et al. reported that berberine inhibited NF-κB signaling, decreased expression of M1 macrophage markers (iNOS, CD86), increased expression of M2 markers (Arg-1, CD206), and promoted a shift from a proinflammatory M1 phenotype toward an anti-inflammatory M2 phenotype. These changes were accompanied by reduced gingival TNF-α and IL-6 levels and increased IL-10 secretion, ultimately attenuating T2DM-exacerbated periodontal inflammation and alveolar bone loss (Mi et al., 2024; Xia et al., 2024).

In addition, berberine can target key inflammatory signaling pathways implicated in the initiation and progression of periodontitis, including NF-κB, MAPK, PI3K/AKT, and AMPK, interrupting inflammatory cascade reactions. In a ligature-induced rat periodontitis model, Gu et al. showed that berberine upregulated expression of the G protein–coupled estrogen receptor (GPR30), inhibited activation of the NF-κB and MAPK pathways, reduced levels of phosphorylated p38 MAPK (p-p38 MAPK) and phosphorylated NF-κB p65 (p–NF-κB p65), decreased gingival secretion of proinflammatory cytokines TNF-α and IL-1β, and increased IL-10 expression, ultimately attenuating inflammatory infiltration and alveolar bone resorption (Gu et al., 2021). Ye et al. reported that Yunvjian decoction (YNJ) containing berberine suppressed periodontal inflammation by inhibiting the NFκB/NLRP3/IL-1β axis and reducing LPS-induced expression of NLRP3 and caspase-1 in rat alveolar bone, limiting apoptosis and IL-1β release (Ye et al., 2024). Moreover, under inflammatory conditions, berberine was found to inhibit the PI3K/AKT pathway, lowering phosphorylated PI3K (p-PI3K) and phosphorylated AKT (p-AKT) levels and reducing IL-1β and IL-6 secretion by RAW264.7 macrophages. Conversely, under osteogenic induction conditions, berberine activated PI3K/AKT signaling and increased expression of osteogenesis-related factors ALP and COL1 in MC3T3-E1 cells. Meanwhile, LY294002, a pharmacological inhibition of PI3K, enhanced the anti-inflammatory effects while attenuating the osteogenic effects (Wang et al., 2023). These findings suggest that berberine does not simply activate or inhibit PI3K/AKT signaling, but rather exerts context-dependent, bidirectional regulation according to cell type and physiological demand, consistent with its pleiotropic, multitarget profile. Berberine activates AMPK signaling in macrophages and enhances efferocytosis of apoptotic neutrophils, limiting the release of proteases, ROS, and proinflammatory mediators associated with secondary necrosis and promoting resolution of inflammation (Bae et al., 2011; Ge et al., 2022).

Berberine mitigates periodontal inflammation by reshaping the cytokine network, suppressing proinflammatory mediators while enhancing anti-inflammatory signaling. A substantial body of *in vitro* and *in vivo* evidence indicates that berberine reduces the expression and release of proinflammatory factors, including TNF-α, IL-1β, IL-6, IL-8, IL-17, RANKL, MMP-2, MMP-9, MCP-1, and PCSK9 (Mohammadian Haftcheshmeh and Momtazi-Borojeni, 2021; Li et al., 2024), while increasing secretion of anti-inflammatory cytokines such as IL-10 (Gu et al., 2021), limiting inflammatory tissue injury. Metabolomic analyses further suggest that, in LPS-stimulated human gingival fibroblasts (HGFs), berberine modulates apoptosis-related signaling to decrease proinflammatory mediator production, inhibits caspase-3 activation, protects HGFs from inflammation-associated damage, and reverses LPS-induced metabolic dysregulation (Zhang et al., 2022a). Using network pharmacology complemented by *in vitro* validation, Li et al. found that berberine inhibited LPS-driven Th17 differentiation and reduced secretion of IL-6, IL-8, and IL-17 (Li et al., 2024). Consistently, in an LPS-stimulated human periodontal ligament cell model, berberine suppressed production of monocyte chemoattractant protein-1 (MCP-1), contributing to attenuation of the inflammatory response (Zhang and Yu, 2012).

In summary, berberine may attenuate periodontal tissue injury and inhibit periodontitis progression by modulating immune-cell function, suppressing proinflammatory signaling pathways, and reprogramming the cytokine network to restore host immune homeostasis.

#### 4.1.3 Berberine promotes tissue repair and periodontal regeneration

An ideal therapeutic goal in periodontitis involves regenerating both periodontal hard tissues (alveolar bone and cementum) and soft tissues (gingiva and periodontal ligament). Berberine may facilitate repair of periodontal tissue damage and promote periodontal regeneration by enhancing osteoblastic and fibroblastic differentiation, inhibiting osteoclast activity, and modulating bone metabolic balance.

Berberine may promote periodontal ligament repair and restore soft-tissue attachment by enhancing osteogenic differentiation of human periodontal ligament stem cells (hPDLSCs) and bone marrow–derived mesenchymal stem cells (BMSCs), as well as stimulating the proliferation of HGFs. Liu et al. reported that berberine activated PI3K/AKT signaling, increased the proliferative capacity of hPDLSCs, elevated the proportion of cells in S and G2/M phases, upregulated osteogenesis-related genes *OCN*, *POSTN*, and *OPN*, and increased protein levels of p-PI3K and p-AKT. These changes were accompanied by enhanced ALP activity and osteogenic differentiation (Liu et al., 2024). Zhang et al. demonstrated that berberine promoted osteogenic differentiation of BMSCs via activation of Wnt/β-catenin signaling, increasing accumulation of total and nuclear β-catenin and upregulating osteogenic genes *OSX*, *COL1*, *ALP*, and *OCN* (Zhang et al., 2019). In addition, in an ovariectomized (OVX) rat model of periodontitis, Jia et al. found that berberine modulated the gut microbiota by increasing the abundance of butyrate-producing bacteria, enhancing butyrate generation, and improving intestinal barrier integrity, which was associated with increased expression of *Runx2* and *OCN* in alveolar bone and reduced alveolar bone resorption (Jia et al., 2019). In ligature-induced rat periodontitis models, berberine increased collagen deposition in gingival tissues, inhibited periodontal tissue degradation, promoted regeneration of gingival epithelium and connective tissue, and reduced probing depth (PD) and clinical attachment loss (AL) (Tu et al., 2013; Yu et al., 2008).

Berberine can also reduce alveolar bone resorption by downregulating osteoclast-related gene expression and inhibiting osteoclast differentiation. In a rat apical periodontitis model, Cui et al. reported that berberine decreased osteoclast number and activity, downregulated *MMP-2* and *MMP-9* expression, and upregulated lysyl oxidase (LOX), contributing to extracellular matrix remodeling and restoration of bone metabolic balance (Cui et al., 2023). In addition, a PLGA microsphere gel loaded with berberine was developed that modulates the RANKL/RANK/OPG axis by increasing *OPG* expression and decreasing *RANKL* levels, resulting in suppression of osteoclast activity. In a rat periodontitis model, this formulation significantly increased bone volume fraction (BV/TV) and trabecular thickness (Tb.Th) (Mi et al., 2024).

### 4.2 Applications of berberine in recurrent aphthous ulcer and oral candidiasis

#### 4.2.1 Anti-inflammatory effects of berberine in recurrent aphthous ulcer

**FIGURE 3:**
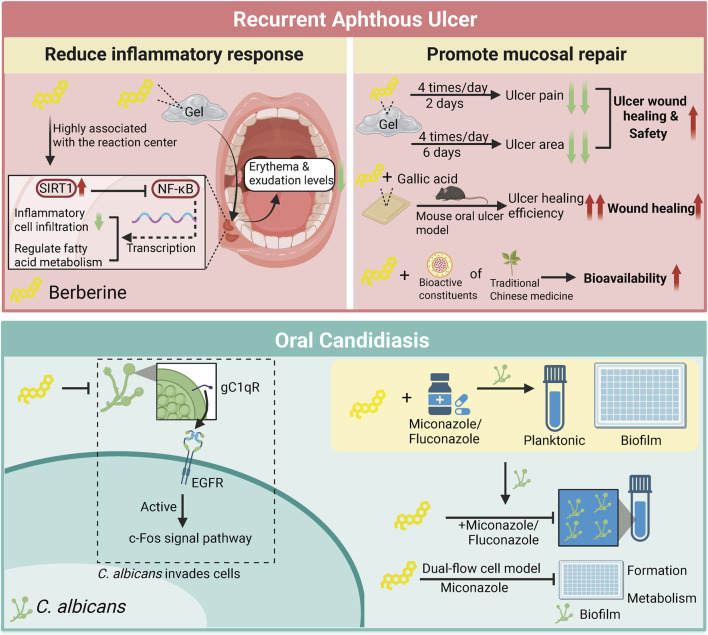
Mechanisms of Berberine in Recurrent Aphthous Ulcer and Oral Candidiasis. Sirtuin 1 (SIRT1), Globular C1q receptor (gC1qR), Epidermal growth factor receptor (EGFR), and *Candida albicans* (*C. albicans*). Created in BioRender.

Recurrent aphthous ulcer represents one of the most common oral mucosal disorders, yet its pathogenesis remains incompletely understood. Currently, evidence largely implicates immune-inflammatory dysregulation in ulcer development (Toche et al., 2007; Al-Samadi et al., 2013). Given its pleiotropic anti-inflammatory properties, berberine has attracted interest as a potential adjunctive therapy for oral ulceration (Figure 3). In mechanistic studies evaluating Zhibai Dihuang Decoction (ZBDHD) for recurrent aphthous ulcer, ZBDHD was shown to upregulate sirtuin 1 (SIRT1) expression, decrease acetylation of NF-κB, and suppress its transcriptional activity, improving oral mucosal histopathologic changes, reducing inflammatory cell infiltration, and modulating fatty acid metabolism. In addition, molecular docking analyses further demonstrated a high degree of complementarity between active constituents in ZBDHD, including berberine, and the active site of SIRT1, suggesting that berberine and related compounds contribute substantially to the therapeutic effects of ZBDHD on oral ulcers by modulating the SIRT1–NF-κB pathway (Shao et al., 2021). In a randomized, double-blind, placebo-controlled clinical trial, Jiang et al. reported that topical application of a berberine-containing gel (5 mg/g) significantly reduced perilesional erythema and exudation in patients with oral ulcers, an effect that may be associated with reduced expression of proinflammatory cytokines (e.g., TNF-α, IL-1β, and IL-6) and immunomodulation through AMPK signaling (Jiang et al., 2013).

#### 4.2.2 Berberine promotes oral mucosal healing

In a randomized, double-blind, placebo-controlled clinical trial conducted by Jiang et al., 84 patients with mild recurrent aphthous ulcers were enrolled and allocated to either a berberine gel group or a placebo group. The assigned gel was applied topically 4 times daily for five consecutive days. The results showed that by day 2 of treatment, ulcer pain scores in the berberine group were already significantly lower than those in the placebo group, and this analgesic benefit persisted through the end of treatment. Moreover, on day 6, the reduction in ulcer area from baseline was significantly greater in the berberine group than in the placebo group, and no participants experienced local irritation or systemic adverse events, supporting the safety of topical berberine and its capacity to promote ulcer wound healing (Jiang et al., 2013). In addition, berberine has been reported to accelerate wound repair by modulating autophagy and stimulating angiogenesis. Accordingly, Issuriya et al. developed a bilayer mucoadhesive patch loaded with Khaolaor Ya-Kwad-Samarn-Lin (KLO), a formulation whose principal bioactive constituents include gallic acid and berberine. Physicochemical testing demonstrated excellent mucosal adhesion and sustained drug-release performance. In a murine oral ulcer model, treatment with the patch increased healing efficiency by 2.78-fold compared with the untreated group and did not produce evidence of local or systemic toxicity (Issuriya et al., 2025).

Evidence suggests that berberine may achieve higher bioavailability when administered as part of a multi-herb formulation combined with other bioactive constituents of traditional Chinese medicine. For example, investigators compared the pharmacokinetics in rats after oral administration of *Coptis* extract alone versus the complete Shuanghua Baishao tablet, a proprietary Chinese medicine used for the management of recurrent aphthous ulcer, and found that the additional herbal components in Shuanghua Baishao tablet facilitated the absorption of five major alkaloids, including berberine, increasing their systemic bioavailability. These findings provide a rationale for designing more efficient compound formulations incorporating berberine for the treatment of recurrent aphthous ulcer (Zhao et al., 2018).

#### 4.2.3 Therapeutic mechanisms of berberine for oral candidiasis

*C. albicans* as the principal causative microorganism, plays a crucial role in initiation and progression of oral candidiasis (Figure 3). With the emergence of antifungal-resistant *Candida* phenotypes, traditional herbal medicines have been proposed as a potential source of novel antifungal strategies (Gharibpour et al., 2021). Increasing evidence indicates that natural constituents from traditional Chinese herbal medicines, including berberine, possess antifungal activity and are particularly effective against *C. albicans* (Zhao et al., 2006). Bao et al. investigated the mechanisms by which Coptidis Rhizoma extract and berberine improve oropharyngeal candidiasis and showed that they target the globular C1q receptor (gC1qR)–epidermal growth factor receptor (EGFR) co-receptor complex, effectively suppressing activation of the downstream ERK/c-Fos signaling pathway and preventing *C. albicans* invasion into host cells, ultimately enhancing therapeutic efficacy (Bao et al., 2024). In addition, studies have evaluated the *in vitro* antifungal activity of berberine alone and in combination with miconazole or fluconazole against both planktonic and biofilm-associated *Candida*. The findings suggest that berberine inhibits the growth of multiple *Candida* species, and that combination therapy with miconazole or fluconazole yields synergistic inhibition of planktonic *C. albicans*. Meanwhile, in a dual-flow cell model that mimics the oral environment, co-treatment with berberine and miconazole markedly suppresses biofilm formation and metabolic activity, providing a potential therapeutic option for biofilm-associated oral candidiasis (Wei et al., 2011).

### 4.3 The application of berberine in dental caries

#### 4.3.1 Berberine inhibits cariogenic bacterial growth

**FIGURE 4:**
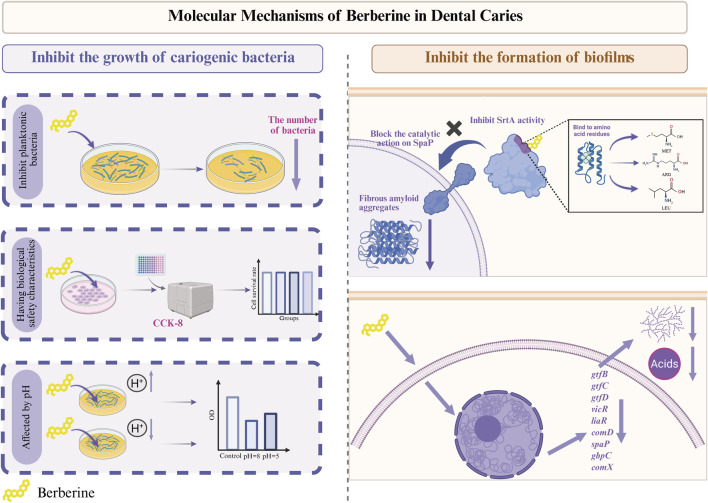
Molecular Mechanisms of Berberine in Dental Caries. Berberine inhibits the growth of cariogenic bacteria (Left panel) and inhibits the formation of biofilms (Right panel). Sortase A (SrtA). Created in BioRender.

Dental caries is a chronic, progressive disease of the dental hard tissues caused by multiple interacting factors, with bacteria as the principal etiologic agent (Selwitz et al., 2007) (Figure 4). *Streptococcus mutans* (*S. mutans*), due to its strong acid production ability and high acid tolerance, is widely regarded as a key cariogenic bacterium and increased abundance of *S. mutans* is closely associated with a markedly elevated risk of caries development and progression (Homayouni Rad et al., 2023). Consequently, berberine, which possesses significant antimicrobial activity, is considered a potential agent for caries prevention and control by inhibiting the growth of cariogenic bacteria. Zeng et al. investigated and evaluated the inhibitory effects of berberine on *S. mutans* and the underlying mechanisms, demonstrating that berberine exerted significant antibacterial activity against planktonic *S. mutans* and killed bacteria by disrupting membrane integrity. Minimum inhibitory concentration (MIC), minimum bactericidal concentration (MBC), and growth-curve analyses further supported a dose-dependent suppression of bacterial proliferation. Importantly, berberine exhibited no cytotoxicity toward several human oral cell types or macrophages (Zeng et al., 2025). In addition, Kazemipoor et al. compared the antibacterial effects of *Berberis vulgaris* extract (with berberine as a major bioactive component) with 2% chlorhexidine (CHX) and ampicillin (10 μg/disc) against cariogenic bacteria, and found that extracts from different parts of *Berberis vulgaris* showed inhibitory activity comparable to that of CHX, indicating that the antibacterial effect of berberine does not differ significantly from that of conventional antimicrobial agents (Kazemipoor et al., 2021). These findings provide supportive evidence for the potential antimicrobial application of berberine. Zhou et al. reported that berberine chloride hydrate exhibited enhanced inhibitory activity against *S. mutans* under alkaline conditions (pH 8) and slightly reduced activity under acidic conditions (pH 5), while maintaining good structural stability and low hemolytic toxicity, further demonstrating that berberine can tolerate intraoral pH fluctuations and retain its antibacterial efficacy (Zhou et al., 2023). Tariq et al. developed an herbal composite formulation containing berberine, tannic acid, eugenol, curcumin, and other constituents, and demonstrated its broad-spectrum antimicrobial activity against dental plaque microorganisms, including microorganisms identified at the genus level as *Streptococcus* spp., *Staphylococcus* spp., and *Candida* spp., with zones of inhibition comparable to those of azithromycin and 0.2% CHX. Topical application also provided rapid hemostasis and analgesia, suggesting that synergistic combinations of berberine with other natural compounds may confer multifunctional benefits—antimicrobial, hemostatic, and analgesic—compared with currently used clinical antimicrobial agents, suggesting potential relevance to the complex oral environment (Tariq et al., 2025).

#### 4.3.2 Berberine inhibits the formation of biofilm

Dental plaque biofilms comprise multispecies microbial communities embedded within an extracellular matrix, which enhances microbial resilience against host immune defenses and antimicrobial agents. Expansion of acidogenic bacteria within the biofilm promotes the development of cariogenic biofilms and ultimately drives dental caries. Mechanistic evidence indicates that berberine chloride hydrate can bind amino acid residues Leu-111, Met-123, and Arg-213 to inhibit the activity of sortase A (SrtA), a central regulator during *S. mutans* biofilm formation, blocking SrtA-mediated catalysis of the surface protein SpaP, reducing the formation of fibrillar amyloid aggregates, and consequently suppressing biofilm development and decreasing enamel demineralization (Zhou Y. et al., 2025). In addition, berberine has been reported to downregulate virulence genes *gtfB*, *gtfC*, *gtfD*, *vicR*, *liaR*, and *comD*, adhesion-associated genes *spaP* and *gbpC*, and the acidogenicity-related gene *comX*, diminishing bacterial adhesion and extracellular polysaccharide synthesis, inhibiting biofilm formation, and further limiting plaque maturation and cariogenicity by reducing acid accumulation (Zhou et al., 2023). Nanomedicine-based delivery systems, characterized by high drug-loading capacity, excellent biocompatibility, controlled release, and the ability for surface modification, enable targeted and stimuli-responsive drug delivery to support precision therapy (Chen et al., 2022). Pourhajibagher et al. developed a berberine-loaded nanoniosomal formulation (nNios@Ber) and demonstrated that, with photodynamic therapy (PDT) as an adjunct, this platform significantly inhibited cariogenic biofilm formation and dose-dependently downregulated *gtfB* expression, supporting further investigation of this berberine-based targeted anti-caries approach (Pourhajibagher et al., 2025).

### 4.4 Applications of berberine in endodontic diseases

**FIGURE 5:**
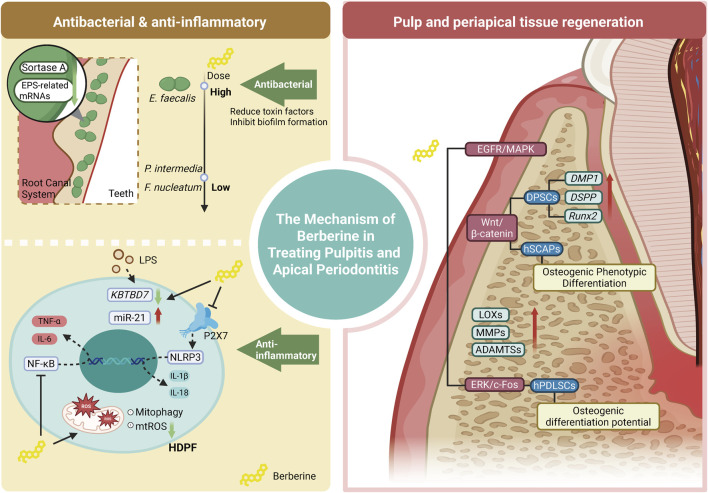
Molecular Mechanisms of Berberine in Pulpitis and Apical Periodontitis. Antibacterial and anti-inflammatory effects (Left) and Pulp and periapical tissue regeneration (Right). *Enterococcus faecalis* (*E. faecalis*), *Prevotella intermedia (P. intermedia)*, *Fusobacterium nucleatum (F. nucleatum),* Mitochondrial reactive oxygen species (mtROS), Human dental pulp fibroblasts (HDPFs), Human periodontal ligament stem cells (hPDLSCs), Lysyl oxidases (LOXs), Matrix metalloproteinases (MMPs), A disintegrin and metalloproteinase with thrombospondin motifs (ADAMTS), and Dental pulp stem cells (DPSCs). Created in BioRender.

Endodontic diseases represent inflammatory disorders primarily triggered by bacterial infection and encompass pulpitis and apical periodontitis. Their pathogenesis involves infection-driven inflammation, excessive immune activation, and cytokine release. Through its broad-spectrum and sustained antimicrobial activity—including eradication of common endodontic pathogens and inhibition of biofilm formation—berberine may effectively control root canal infection (Figure 5) (Marques et al., 2024).

#### 4.4.1 Berberine as an intracanal medicament and root canal irrigant

In the management of endodontic diseases, berberine has been investigated primarily as an intracanal medicament and root canal irrigant. With respect to intracanal disinfection, a clinical study by Su reported that berberine achieved superior antibacterial efficacy for disinfection of infected primary teeth compared with conventional agents such as formocresol (FC) and camphorated phenol (CP). It showed no evident toxic side effects and was characterized by favorable biocompatibility and broad-spectrum antimicrobial activity. These findings support its potential as a safe and effective alternative for clinical intracanal disinfection (Su, 1992). In addition, Marques et al. developed a PLGA-based nanodelivery system that significantly enhanced antimicrobial performance within the root canal system. Berberine-loaded nanoparticles markedly reduced the biofilm activity of *Enterococcus faecalis* (*E. faecalis*) and *C. albicans* within 24 h while demonstrating good biosafety (Marques et al., 2024). In endodontic therapy, berberine also has emerged as a potential irrigant: when combined with low-concentration CHX, it exerts synergistic clearance against mixed-species biofilms and *E*. *faecalis*, and exhibits lower cytotoxicity than higher concentrations of NaOCl, suggesting its potential as an adjunctive or alternative irrigation strategy (Xie et al., 2012).

#### 4.4.2 Targeting persistent endodontic biofilms and refractory infection

Endodontic infections are commonly driven by polymicrobial biofilms, with *E. faecalis* frequently implicated as a representative pathogen. Available evidence indicates that berberine exhibits notable antimicrobial effect against common endodontic pathogens such as *Prevotella intermedia* and *F*. *nucleatum*, and that higher concentrations of berberine can also achieve inhibitory effects against *E. faecalis* (Xie et al., 2012), underscoring its broad-spectrum antimicrobial profile and therapeutic potential in pulp disease.

The therapeutic potential of berberine in this context appears to stem largely from its capacity to disrupt bacterial virulence determinants and inhibit biofilm formation, limiting the initiation and progression of pulpal and periapical disease. *E. faecalis* can persist within the root canal system as a biofilm and is considered a key contributor to refractory apical periodontitis and periapical bone resorption (Alghamdi and Shakir, 2020; Stuart et al., 2006). Experimental evidence indicates that berberine downregulates *E. faecalis srtA* (sortase A) and extracellular polysaccharide (EPS)-associated mRNA expression, inhibiting biofilm development and disrupting established biofilms (Alghamdi and Shakir, 2020; Stuart et al., 2006; Mori et al., 2023).

#### 4.4.3 Regulation of host tissue responses and repair in pulpal and periapical lesions

Beyond infection control, berberine may also regulate host tissue responses in pulpal and periapical lesions. In LPS–stimulated human dental pulp fibroblasts (HDPFs), berberine has been shown to upregulate miR-21 and downregulate *KBTBD7* expression, inhibiting NF-κB pathway and reducing the expression of proinflammatory cytokines such as IL-6 and TNF-α (Song et al., 2020). Meanwhile, berberine can inhibit NLRP3 inflammasome activation and decrease the release of IL-1β and IL-18 through mechanisms that include induction of mitophagy, reduction of mitochondrial reactive oxygen species (mtROS) generation, and interference with P2X7 receptor pathway (Jiang et al., 2015; Wang et al., 2021b; Tian et al., 2021; Liu et al., 2020; Zhao et al., 2022; Vivoli et al., 2016). These effects may help attenuate inflammation-associated tissue damage and create a microenvironment favorable for pulpal and periapical repair.

Evidence further suggests that berberine promotes the proliferation of dental pulp fibroblasts and enhances stem cell differentiation toward osteoblastic and odontoblastic phenotypes, supporting pulp–dentin complex repair and periapical tissue regeneration (Song et al., 2020; Kyaw et al., 2025). Mechanistically, berberine promotes nuclear translocation of β-catenin and upregulates osteogenic and odontogenic genes, including *DMP1*, *DSPP*, and *Runx2*, in dental pulp stem cells (DPSCs), while also activating EGFR/MAPK signaling and enhancing osteogenic differentiation and mineralized nodule formation. In human stem cells from the apical papilla (hSCAPs), berberine activates Wnt/β-catenin signaling to promote osteogenic differentiation and root remodeling. In addition, berberine may contribute to extracellular matrix remodeling and local bone metabolic balance by increasing LOX expression and downregulating MMPs and a disintegrin and metalloproteinase with thrombospondin motifs (ADAMTS) family members (Cui et al., 2023; Su, 1992; Xie et al., 2012; Liu et al., 2025a).

### 4.5 Therapeutic Mechanisms of Berberine in Oral Tumors

#### 4.5.1 Berberine inhibits the proliferation of tumor cells

**FIGURE 6:**
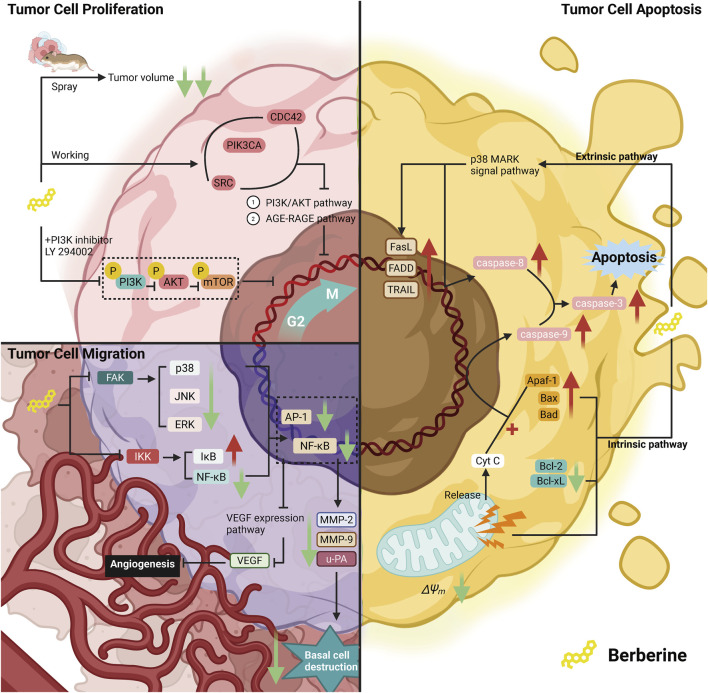
Therapeutic Mechanisms of Berberine in Oral Tumors. Tumor cell proliferation, apoptosis, and migration. Fas-associated death domain protein (FADD), Fas ligand (FasL), TNF-related apoptosis-inducing ligand (TRAIL), Vascular endothelial growth factor (VEGF), Matrix metalloproteinase (MMP)-2, MMP-9, and urokinase-type plasminogen activator (u-PA). Created in BioRender.

Head and neck malignancies predominantly comprise oral squamous cell carcinoma (OSCC) and laryngeal squamous cell carcinoma (LSCC), which exhibit aggressive progression driven largely by aberrant proliferation of tumor cells. Berberine has been shown to suppress the proliferative activity of oral cancer cells by modulating key signaling pathways, inducing cell-cycle arrest, and regulating oncogenic programs (Figure 6). Using network pharmacology, Liu et al. identified SRC, PIK3CA, and CDC42 as core targets of berberine, enriched in proliferation-related pathways such as PI3K/AKT and AGE–RAGE. Molecular docking indicated excellent binding (binding energies < −5 kcal/mol) with good stability, and subsequent *in vitro* experiments demonstrated that berberine reduced protein levels of RAGE, p-PI3K, p-AKT, and p-mTOR, inhibiting OSCC cell proliferation via suppression of the PI3K/AKT/mTOR axis (Liu et al., 2025a). In LSCC, berberine similarly inhibited proliferation and viability of SNU-899 and AMC-HN-8 cells by inhibiting PI3K/AKT/mTOR signaling pathway, and co-treatment with the PI3K inhibitor LY294002 further enhanced the inhibitory effect of berberine (Lin et al., 2024). Beyond pathway inhibition, berberine can restrain proliferation by inducing cell-cycle arrest: Li et al. reported an IC50 of 218.52 ± 18.71 μM for berberine hydrochloride in the OSCC cell line Tca8113 (MTT assay), and flow cytometry showed G2/M-phase arrest following berberine treatment, consistent with impaired cell-cycle progression (Li Q. et al., 2023). In a hamster oral cancer model, Tamane et al. formulated a standardized *Berberis* extract containing berberine as a buccal spray (SBAE-BS) and demonstrated that SBAE-BS significantly reduced tumor volume (*p* = 0.0345) with no organ toxicity observed, supporting the ability of berberine-containing preparations to inhibit oral tumor growth *in vivo* (Tamane et al., 2023).

#### 4.5.2 Berberine induces the apoptosis of tumor cells

Apoptosis evasion represents an important hallmark of oral tumor cells, and berberine has been reported to trigger apoptotic cell death by engaging both the mitochondrial (intrinsic) and death receptor (extrinsic) pathways while regulating apoptosis-associated proteins, showing partial selectivity toward tumor cells. Berberine can simultaneously activate the death receptor–mediated extrinsic pathway and the mitochondria-mediated intrinsic pathway. In the extrinsic pathway, berberine activates p38 MAPK signaling and increases the expression of Fas ligand (FasL), Fas-associated death domain protein (FADD), and TNF-related apoptosis-inducing ligand (TRAIL), accompanied by upregulation of caspase-8. In the intrinsic pathway, berberine decreases the antiapoptotic proteins Bcl-2 and Bcl-xL while increasing proapoptotic proteins Bax, Bad, Bak, and Apaf-1, leading to the loss of mitochondrial membrane potential, the release of cytochrome c, the formation of the apoptosome, and the subsequent activation of caspase-9. Caspase-8 and caspase-9 converge on caspase-3 activation, triggering the downstream apoptotic cascade and culminating in tumor cell apoptosis (Lin et al., 2007; Kim et al., 2015). In addition, berberine has been shown to increase intracellular ROS and cytosolic Ca^2+^ levels, promoting mitochondrial depolarization and further engaging the intrinsic apoptotic program. Berberine also upregulates apoptosis-inducing factor (AIF) and endonuclease G (EndoG) expression and induces apoptosis in tumor cells (Ho et al., 2009a; Skonieczna et al., 2019).

#### 4.5.3 Berberine inhibits the invasion and metastasis of tumor cells

Tumor invasion and metastasis serve as key determinants distinguishing malignant from benign neoplasms and involve coordinated processes such as angiogenesis, tumor cell adhesion, and degradation of the ECM. Berberine has been reported to suppress angiogenesis and ECM destruction in oral malignancies by downregulating VEGF and MMPs and by inhibiting relevant signaling pathways. In the head and neck squamous cell carcinoma (HNSCC) cell line FaDu, Seo et al. showed that berberine markedly reduced expression of VEGF, MMP-2, and MMP-9, limiting endothelial cell proliferation and ECM degradation and ultimately inhibiting angiogenesis (Seo et al., 2015). Mechanistic analyses further indicated that berberine suppresses VEGF and MMP production by inhibiting MAPK, PI3K/AKT, and NF-κB signaling, which contributes to reduced invasive and metastatic potential. Specifically, berberine downregulated focal adhesion kinase (FAK) signaling, decreased levels of key phosphorylated MAPK proteins (p-p38, p-JNK, and p-ERK), and inhibited AKT phosphorylation. Meanwhile, it reduced the NF-κB activator IKK while increasing the NF-κB inhibitor IκB, suppressing NF-κB transcriptional activity and downregulating invasion-associated effectors, including MMP-2, MMP-9, and urokinase-type plasminogen activator (u-PA), ultimately limiting tumor cell–mediated degradation of the ECM and basement membrane (Seo et al., 2015; Ho et al., 2009b).

Table 1 highlights some key study types, experimental models, preparations, doses and durations, and primary outcomes of berberine-based interventions in periodontitis, oral mucosal diseases, dental caries, endodontic diseases, and oral cancers (Table 1).

**TABLE 1:** Summary of berberine and its formulations in oral disease studies.

Disease type	Study type	Experimental model	Preparation	Dose and duration	Primary outcome	Refs
Periodontitis	*In vitro* and *in vivo*	*P. gingivalis,* HGECs, HGF; rat model of periodontitis	BiCD-Ber encapsulated in hyaluronic acid hydrogel	BiCD-Ber MIC 41.7 μg/mL; hydrogel 1–5 mg/mL; 21 days	Inhibited *P. gingivalis* and gingipain; reduced alveolar bone loss	Li X. et al. (2025)
Periodontitis	*In vitro*	RAW264.7 and MC3T3-E1 osteoblasts	Berberine loaded in thermosensitive hydrogel	Berberine 5 μM; 7 days	Inhibited PI3K/AKT/NF-κB; upregulated ALP, COL-1, OCN, Runx-2; promoted osteogenesis	Wang et al. (2023)
Aphthous Ulcer	Clinical trial	Patients with minor recurrent aphthous stomatitis	Berberine gelatin	5 mg/g berberine gel applied topically 4 times daily; 5 days	Reduced ulcer pain, ulcer size, erythema and exudation	Jiang et al. (2013)
Aphthous Ulcer	*In vivo*	Rat model of oral ulcer	Polyherbal KLO4 extract (contains berberine) in bilayer patch	0.0625 g extract/patch; 7 days	2.78-fold higher healing rate than untreated	Issuriya et al. (2025)
Oral Candidiasis	*In vitro* and *in vivo*	FaDu cells; mouse model of oropharyngeal candidiasis	CRE; berberine	CRE 50–200 mg/kg; berberine 10 μM; 4 days	Protected epithelial barrier; inhibited gC1qR-EGFR/ERK/c-fos; alleviated OPC	Bao et al. (2024)
Dental caries	*In vitro*	*S. mutans,* oral epithelial cells, macrophages	Berberine hydrochloride	50–400 μg/mL; 1 day	Inhibited planktonic growth, biofilm formation, acid production; downregulated *gtfB*/*ldh*/*vicR*	Zeng et al. (2025)
Dental caries	*In vitro*	*S. mutans*, *S. sobrinus*, *S. sanguinis*, *L. rhamnosus*	*Berberis vulgaris* stem/fruit extracts (contains berberine)	1 mg per disk; 2 days	Antibacterial activity comparable to chlorhexidine and ampicillin	Kazemipoor et al. (2021)
Dental caries	*In vitro*	*S. mutans* UA159 at pH 5, 7.2, 8	BH	32, 64, 128, 256, 512 μg/mL; 1 day	Inhibited biofilm; downregulated *srtA*/*spaP*/*gbpC*/*comX*/*ldh*; effect pH-dependent	Zhou et al. (2023)
Endodontic diseases	*In vitro*	*E. faecalis*; *C. albicans*; HGF	Berberine loaded in PLGA nanoparticles	Berberine nanoparticles 10–40 μg/mL; berberine≥125 μg/mL; 1 day	Nanoparticles at 20, 30, 40 μg/mL inhibited pathogens (free berberine required ≥125 μg/mL)	Marques et al. (2024)
Apical Periodontitis	*In vivo*	Rat model of apical periodontitis	Berberine	2 mg/mL; 6 weeks	Downregulated MMPs; reduced apical lesion and alveolar bone loss	Cui et al. (2023)
Oral Tumors	*In vitro*	SCC-9 cells	Berberine	0–80 μM; 1–3 days	Inhibits RAGE/PI3K/AKT/mTOR; inhibit proliferation and migration	Liu et al. (2025a)
Oral Tumors	*In vitro*	Various human cancer cell lines (OSCC, nasopharyngeal, breast, cervical, colon)	Berberine hydrochloride	IC_50_: Tca8113 218.5 μM; 0–36 h	Inducing cell apoptosis and cell cycle arrest; upregulated BAX, downregulated BCL-2	Li Q. et al. (2023)
Oral Tumors	*In vitro* and *in vivo*	KB cells; hamster model of oral cancer	Standardized *Berberis aristata* extract (contains berberine) buccal spray	Berberine 0.6 mg/mL 4 weeks	Cytotoxic and chemo-protective effects	Tamane et al. (2023)
Oral Tumors	*In vitro*	SCC-25 cells	BC and resveratrol	10 μg/mL; 1 day	Increased intracellular ROS; synergistic effect	Skonieczna et al. (2019)

BiCD-Ber: berberine conjugated with bismuth-doped carbon dots; CRE: coptidis rhizoma extract; BH: berberine chloride hydrate; PLGA: poly (lactic-co-glycolic acid); BC: berberine chloride; OPC: oropharyngeal candidiasis; HGECs: human gingival epithelial cells; HGF: human gingival fibroblasts; OSCC: oral squamous cell carcinoma; *P. gingivalis*: *Porphyromonas gingivalis*; *S. mutans*: *Streptococcus mutans*; *S. sobrinus*: *Streptococcus sobrinus*; *S. sanguinis*: *Streptococcus sanguinis*; *L. rhamnosus*: *Lactobacillus rhamnosus*; *E. faecalis*: *Enterococcus faecalis*; *C. albicans*: *Candida albicans*.

## 5 Physicochemical and pharmacokinetic limitations of berberine

### 5.1 Physicochemical properties of berberine

Berberine is a quaternary isoquinoline alkaloid whose active cationic form has the molecular formula C_20_H_18_NO_4_^+^ and a molecular weight of 336.36 g/mol. It is commonly used as the chloride salt, berberine chloride, also known as berberine hydrochloride, with the molecular formula C_20_H_18_ClNO_4_ and a molecular weight of 371.81 g/mol. The physicochemical properties discussed below mainly refer to pure berberine or berberine hydrochloride unless otherwise specified. Berberine, particularly in its commonly used chloride salt form, has been described as having poor aqueous solubility, poor liposolubility, and low membrane permeability. In simulated physiological media, the solubility of a standard berberine formulation was reported to be 1.65 ± 0.01 mg/mL in water at pH 7.0, 5.42 ± 0.37 mg/mL in simulated intestinal solution at pH 6.8, and 0.02 ± 0.01 mg/mL in simulated gastric solution at pH 1.2. Its limited permeability has also been demonstrated in Caco-2 monolayers, with an apparent permeability coefficient of 4.93 × 10^−6^ ± 4.28 × 10^−7^ cm/s for standard berberine (Solnier et al., 2023), and in rat jejunal Ussing chamber experiments, with a Papp value of 8.22 ± 0.14 × 10^−7^ cm/s (He et al., 2023). These physicochemical limitations, together with formulation-dependent dissolution and solid-state stability, contribute to the poor gastrointestinal absorption of berberine.

### 5.2 Pharmacokinetic limitations of berberine

**FIGURE 7:**
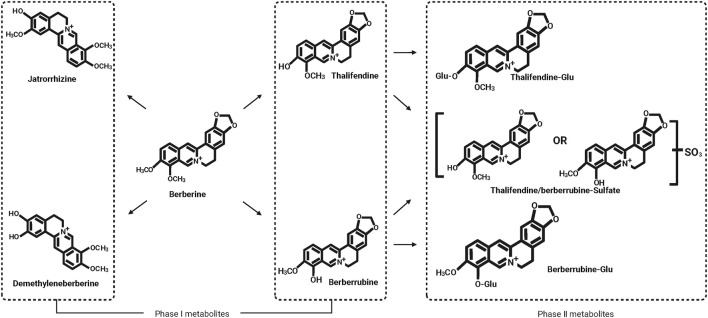
Chemical structures of major berberine metabolites after oral administration of berberine or berberine formulations. Glu: glucuronide. Redrawn based on Ref (Kwon et al., 2020). and created with BioRender.

The pharmacokinetic limitations of berberine are mainly reflected in its limited intestinal absorption and extremely low systemic exposure after oral administration. In rats, the oral bioavailability of berberine has been reported to be approximately 0.68%, and nearly 99.5% of an oral dose may be lost during gastrointestinal first-pass elimination (Chen et al., 2011; Liu et al., 2010; Marques et al., 2023). This poor oral exposure is mainly associated with extensive intestinal metabolism and P-glycoprotein (P-gp)-mediated efflux. Several metabolites have been identified in rat biological samples, including phase I metabolites such as berberrubine, thalifendine, demethyleneberberine, and jatrorrhizine, as well as phase II conjugates such as thalifendine-glucuronide, berberrubine-glucuronide, and thalifendine/berberrubine-sulfate (Kwon et al., 2020). The chemical structures of berberine and its major metabolites are shown in Figure 7.

### 5.3 Physicochemical modification strategies to overcome the pharmacokinetic limitations of berberine

At present, physicochemical modification of berberine mainly aims to improve its solubility, dissolution behavior, membrane permeability, and systemic exposure, overcoming its pharmacokinetic limitations. Cocrystals constructed with cocrystal formers including gentisic acid, gallic acid, and pamoic acid can alter the solid-state properties of berberine, resulting in significant increases in solubility (up to 2-fold), dissolution, permeability (up to 5.98-fold), and peak plasma concentration (Sai Durga Surampudi et al., 2025). A novel cocrystal of berberine with 3-methylcinnamic acid increased solubility in polar solvents by 15.8-fold, increased the intestinal absorption rate constant by 5.6-fold, and reduced hygroscopicity (He et al., 2023). In addition, formation of salt/ion-pair complexes can improve lipophilicity and membrane permeability, increasing relative bioavailability by more than 4-fold (Chen et al., 2024). Food-grade formulations, including a berberine–phospholipid complex (Berberine Phytosome®) and a lipid micellar preparation (Berberine LipoMicel®), have been reported in human studies to increase berberine solubility, permeability, and oral bioavailability by up to 10-fold and 6-fold, respectively, without adverse events (Solnier et al., 2023; Petrangolini et al., 2021).

## 6 Safety profile and therapeutic interactions of berberine

### 6.1 Systemic safety and local intraoral toxicity of berberine

Available clinical evidence suggests that berberine generally has an acceptable systemic safety profile after oral administration, although direct evidence regarding local intraoral toxicity remains limited. Umbrella-review and placebo-controlled trial data indicate that the most reported adverse events are mild-to-moderate gastrointestinal symptoms, including constipation, diarrhea, nausea, abdominal discomfort, and bloating (Li Z. et al., 2023; Liu et al., 2025b). In a recent meta-analysis of randomized placebo-controlled trials (Liu et al., 2025b), berberine did not significantly increase the risk of overall adverse events, gastrointestinal adverse events, or stool abnormalities compared with placebo. Several trials also reported no clinically relevant abnormalities in liver or kidney function parameters. Notably, oral or dental adverse events were rarely reported, and one trial recorded dental and oral disorders only in the control group, not in the berberine group. Therefore, while systemic tolerability appears generally favorable, dedicated studies using oral mucosal cells, gingival fibroblasts, periodontal ligament cells, and long-term intraoral exposure models are still needed to define the local safety profile of berberine-based oral formulations.

### 6.2 Therapeutic interactions and adjunctive effects of berberine

Beyond safety concerns, berberine-related interactions with fluconazole, miconazole, gut microbiota, and phototherapy also merit consideration. Regarding antifungal agents, berberine can potentiate the activity of azoles against *C. albicans*, including fluconazole-resistant strains. In combination with fluconazole, this synergism has been associated with impaired mitochondrial function, reduced intracellular ATP production, inhibition of ATP synthase activity, and increased endogenous ROS accumulation, while combination with miconazole has been shown to markedly inhibit *C. albicans* biofilm formation and reduce the metabolic activity of preformed biofilms (Wei et al., 2011). Berberine has bidirectional interactions with the gut microbiota. Gut microbial nitroreductases can convert berberine into dihydroberberine, which is more readily absorbed and can be oxidized back to berberine in intestinal tissues, thereby improving berberine absorption. Conversely, changes in gut microbiota caused by antibiotics, probiotics, diet, or dysbiosis may modify berberine bioavailability by affecting microbial metabolism, bile acid transformation, enterohepatic recirculation, and intestinal transporters (Zhang et al., 2021). Beyond drug- and microbiota-related interactions, berberine may also interact beneficially with phototherapy by serving as a natural photosensitizer in PDT, which requires a photosensitizer, light of an appropriate wavelength, and molecular oxygen. Owing to its photoactive properties, berberine can be activated by blue light within its absorption range, such as 405 ± 10 nm blue laser irradiation, leading to the generation of ROS, including singlet oxygen and free radicals. These ROS induce oxidative stress, disrupt microbial membranes and metabolic pathways, and enhance antimicrobial and antibiofilm activity (Pourhajibagher et al., 2025; Marques et al., 2023). In dental caries, this photodynamic mechanism may contribute to the suppression of cariogenic *S. mutans* biofilms, supporting berberine-mediated PDT as a potential adjunctive anti-caries strategy (Pourhajibagher et al., 2025).

## 7 Novel drug-delivery systems for berberine in oral disease therapy

In recent years, novel drug-delivery systems have emerged as a major research focus because they can enhance drug solubility, enable controlled and sustained release at the site of action, prolong drug residence time, improve targeting, and reduce cytotoxicity (Zhang et al., 2022b). Such platforms can be designed not only for local intraoral delivery but also to enhance systemic exposure after oral administration. Common delivery systems include nanoparticles/microspheres, hydrogels, and liposomes. Nanocarrier-based delivery can address limitations such as poor solubility, inadequate absorption, low permeability, size-related constraints, instability, and extensive first-pass metabolism. Although it has been explored extensively in oncology, such as ovarian cancer, its application in oral cancers and other oral diseases remains largely preclinical and requires further validation (Zhang et al., 2023). Hydrogels, which consist of three-dimensional hydrophilic polymer networks capable of absorbing and retaining large amounts of water or biological fluids, can adhere to both soft and hard tissues and facilitate localized and controllable drug delivery—for example, direct placement into periodontal pockets for periodontitis treatment—reducing systemic adverse effects while improving therapeutic efficacy (Pan et al., 2025).

### 7.1 Nanoformulation-based delivery strategies for berberine

#### 7.1.1 Characterization of berberine-based nanoformulations

Hydrodynamic size, zeta potential, and polydispersity index (PDI) are key physicochemical parameters for evaluating berberine-based nanoformulations. Hydrodynamic size directly affects mucosal permeation, biofilm penetration, cellular uptake, and local retention. In oral applications, berberine nanocarriers are generally designed within the nanometer range to balance tissue penetration with residence time. For example, PLGA-based berberine nanoparticles have been reported to exhibit particle sizes of approximately 150–200 nm, facilitating penetration through dental plaque biofilms (Mi et al., 2024), whereas chitosan-based berberine nanoparticles are typically larger, approximately 200–300 nm, which may favor mucoadhesion through interactions with negatively charged mucins (Khan et al., 2023). Zeta potential reflects the surface charge of nanoparticles and is closely related to colloidal stability, aggregation tendency, and biological interactions. Positively charged systems, such as chitosan-based nanoparticles or cationic liposomes, can enhance mucoadhesion and prolong local retention through electrostatic interactions with mucosal surfaces and biofilm matrices (Khan et al., 2023; Mahmoud et al., 2022), whereas neutral or negatively charged formulations, such as some PLGA-based nanoparticles, may reduce nonspecific binding and facilitate penetration into mucus or deeper biofilm structures (Marques et al., 2024). PDI serves as a dimensionless parameter that quantifies the breadth of particle size distribution within a nanoparticle formulation. A low PDI value, typically below 0.3, signifies a homogeneous particle population. Several berberine-loaded nanoformulations, including PLGA nanoparticles and chitosan-based nanoparticles, have achieved low PDI values, such as 0.14 and 0.236, respectively, suggesting favorable formulation uniformity and reproducibility (Saha et al., 2024; Saleh et al., 2021). Therefore, coordinated optimization of hydrodynamic size, zeta potential, and PDI is essential for improving the colloidal stability, mucosal retention, biofilm penetration, and drug-release consistency of berberine-based nanoformulations in oral disease therapy (Duong et al., 2021; Nguyen et al., 2022; Nguyen et al., 2023; Wu et al., 2024).

#### 7.1.2 Comparator design for evaluating berberine-based nanoformulations

Clear comparator design is essential for evaluating the therapeutic contribution of berberine-based nanoformulations. In principle, studies should include free berberine, the corresponding berberine-loaded nanoformulation, and a blank vehicle, together with untreated or disease-model controls when appropriate. Comparison with free berberine helps determine whether the nanoformulation improves drug performance, including solubility, stability, bioavailability, mucosal retention, biofilm penetration, or pharmacological efficacy. Blank vehicle controls are equally important because carriers such as liposomes, chitosan-based nanoparticles, hydrogels, or polymeric matrices may themselves exhibit mucoadhesive, antimicrobial, or immunomodulatory effects. For example, the BiCD-Ber hydrogel study compared BiCD, Ber-NH_2_, BiCD-Ber, and BiCD-Ber-loaded hydrogel systems, helping to distinguish the effects of the carbon-dot carrier, berberine functionalization, and hydrogel-mediated local delivery (Li X. et al., 2025). Overall, well-defined comparator groups improve mechanistic interpretability and clarify whether the observed benefits arise from berberine itself, nanocarrier-assisted delivery, or the intrinsic biological activity of the vehicle.

#### 7.1.3 Release profile and intraoral stability of berberine-based nanoformulations

The release profile and intraoral stability of berberine-based nanoformulations are essential for achieving sustained local therapy in oral diseases. Berberine-loaded nanocarriers often exhibit a biphasic release pattern, with an initial burst release that provides rapid drug availability, followed by a sustained phase governed by diffusion or carrier degradation. For example, berberine-loaded nanostructured lipid carriers showed controlled release over 24 h (Deng et al., 2020), while chitosan/alginate nanogels displayed pH-responsive sustained release under acidic conditions relevant to inflamed oral tissues (Singh et al., 2024). Intraoral stability is equally important because salivary flow, enzymatic activity, pH fluctuations, and oral microbiota may affect nanoparticle integrity and drug retention. Stability assessments therefore commonly monitor particle size, PDI, zeta potential, morphology, and drug leakage in simulated saliva or biological fluids. Strategies such as mucoadhesive surface modification with chitosan or hyaluronic acid and incorporation of stabilizing excipients, including alginate or cyclodextrins, may enhance resistance to salivary washout, reduce premature drug leakage, and prolong local residence time (Singh et al., 2024; Khan et al., 2022). Therefore, optimizing both release kinetics and intraoral stability is critical for improving the formulation performance and local applicability of berberine-based nanoformulations in oral disease therapy (Deng et al., 2020; Singh et al., 2024; Khan et al., 2022; Zheng et al., 2023; Eskandrani et al., 2026; Zhou et al., 2026).

#### 7.1.4 Nanocarrier-based strategies to improve the pharmacokinetic performance of berberine

Nanocarrier-based systems, including solid lipid nanoparticles (SLNs) (Nguyen et al., 2023), liposomes (Kutbi et al., 2021), bile salt–containing vesicles (Elkomy et al., 2022), polymeric nanoparticles, and mixed micelles (Kwon et al., 2020), can improve the oral bioavailability of berberine by enhancing solubility, stability, and intestinal uptake. Using a spray-drying approach, investigators prepared berberine-loaded SLNs with a uniform particle size and an encapsulation efficiency of approximately 90% and demonstrated stable sustained-release behavior. *In vitro* assays showed markedly improved stability and absorption, suggesting the potential to enhance the oral performance of berberine (Nguyen et al., 2023). Similarly, bile salt–containing nanovesicles increased the relative bioavailability of berberine by 6.4-fold compared with a solution formulation while also improving stability and sustained-release characteristics (Elkomy et al., 2022). A chitosan–alginate nanoparticle prepared via ionic gelation improved intestinal permeability and intracellular retention, resulting in a > 4-fold increase in oral bioavailability in rats (Kohli et al., 2021). Hyaluronic acid–modified berberine liposomes have been developed and, by virtue of their lipophilicity and small particle size, can facilitate intestinal permeation, provide sustained release for up to 24 h, and significantly increase oral bioavailability in rats (Kutbi et al., 2021). In addition, a Brij-modified chitosan nanocarrier has been shown to transiently modulate tight junctions and inhibit P-gp–mediated efflux, resulting in a 4.4-fold increase in intestinal permeability and oral bioavailability of berberine compared with the free drug (Xiong et al., 2021).

### 7.2 Hydrogel-based local delivery platforms for berberine

Hydrogel-based local delivery platforms have also been developed to further enhance berberine retention, sustained release, and targeted antimicrobial efficacy. One group engineered a berberine-loaded chitosan/carboxymethyl-β-cyclodextrin hydrogel (CS/CMCD hydrogel), which simultaneously increased berberine solubility and enabled sustained release. Experimental data supported robust antibacterial activity and favorable biosafety (Clemence et al., 2024). To improve berberine bioavailability and targeting within the hypoxic, difficult-to-access periodontal pocket, investigators incorporated BiCD-Ber into a hydrogel matrix. The positive charge of Ber-NH_2_ enhanced bacterial binding and facilitated intracellular entry of bismuth ions (Bi^3+^), which inhibit bacterial metabolic enzymes by interacting with thiol (–SH) and amino (–NH_2_) groups, acting synergistically with berberine through combined membrane disruption and enzyme inhibition to substantially increase antibacterial efficiency. Consequently, this platform enhanced berberine-mediated clearance of *P. gingivalis*, neutralized its virulence factors, restored host immune responses, and preserved epithelial barrier integrity, effectively preventing alveolar bone resorption (Li X. et al., 2025).

## 8 The advantages and limitations of application of berberine in oral diseases

Berberine offers several distinctive advantages for the treatment of oral diseases. First, its natural origin is associated with favorable biocompatibility and low toxicity: *in vitro* studies have shown no detectable cytotoxicity toward normal oral cells such as human gingival fibroblasts and oral epithelial cells, and clinically applied formulations (e.g., gels and sprays) have not demonstrated significant adverse effects. Second, its multiple effects enable simultaneous targeting of several pathogenic components of oral diseases. For example, in periodontitis, berberine can provide combined antibacterial and anti-inflammatory effects while supporting alveolar bone regeneration, potentially reducing the need for multi-drug therapies. Third, mechanistic diversity allows adaptation to distinct disease phenotypes, with antibacterial effects mediated by disruption of bacterial membranes and suppression of virulence-gene expression, and antitumor and anti-inflammatory effects mediated through regulation of apoptotic pathways and immune cell polarization. Fourth, berberine has been developed into various topical formulations, including gels, oral patches, and buccal sprays, to meet the practical requirements of intraoral delivery, such as ease of use and direct application to diseased sites.

Despite these encouraging findings, several limitations must be addressed before berberine can be more broadly implemented for oral disease management. First, berberine has poor aqueous solubility and limited lipophilicity, resulting in limited bioavailability. Second, much of the evidence supporting berberine in oral diseases is limited to *in vitro* studies and animal models. Available clinical investigations are typically small in scale and short in duration. This underscores the need for adequately powered, long-term randomized clinical trials to confirm efficacy, durability, and safety of berberine in humans.

## 9 Summary and prospectives

Berberine, a bioactive isoquinoline alkaloid isolated from traditional Chinese medicinal herbs such as *Coptis* species, exhibits pleiotropic pharmacologic activities—including antibacterial, anti-inflammatory, antioxidant, and antitumor effects—and substantial progress has been made in exploring its applications for oral diseases. In periodontitis, berberine suppresses the growth of periodontal pathogens such as *P. gingivalis* and *F*. *nucleatum*, reduces secretion of proinflammatory cytokines including TNF-α, IL-1β, and IL-6, and modulates signaling pathways such as PI3K/AKT and NF-κB, providing coordinated antibacterial and anti-inflammatory effects with potential benefits for alveolar bone regeneration (Zhou W. et al., 2025). In recurrent aphthous ulcer and apical periodontitis, berberine has shown effects on accelerating wound healing, modulating bone metabolism, and attenuating inflammatory responses (Jiang et al., 2013; Song et al., 2020). In dental caries, berberine inhibits *S. mutans* biofilm formation, blocks sortase A activity, and downregulates virulence genes such as *gtfB*, reducing enamel demineralization and supporting a clear anti-caries potential (Zhou et al., 2023; Zhou Y. et al., 2025). Moreover, in head and neck cancers (e.g., OSCC and LSCC), berberine can induce tumor cell apoptosis and arrest the cell cycle at G2/M, while inhibiting proliferation and migration through suppression of PI3K/AKT/mTOR, MAPK, and NF-κB pathways (Liu et al., 2025a). Collectively, research on berberine has progressed from *in vitro* studies and animal models toward early clinical evaluation, providing a foundation for further translational development.

Moreover, the application of berberine in oral diseases is advancing toward greater precision, improved efficacy, and formulation-driven innovation. To address its poor aqueous solubility and limited bioavailability, a growing range of delivery platforms—including nanocarriers (e.g., nanovesicles and bismuth-doped carbon dots), hydrogels, and mucoadhesive patches—has been developed to enhance berberine stability and targeting while enabling sustained and controlled release. For example, blue-light, which activated berberine nanocarriers can augment photodynamic therapy, mediated disruption of cariogenic biofilms (Pourhajibagher et al., 2025). From a therapeutic perspective, combination approaches are gaining prominence. When used in conjunction with BMP9 (Zhou S. et al., 2025), probiotics (Li S. et al., 2025), or photodynamic therapy (Pourhajibagher et al., 2025), berberine may achieve multi-target synergy that amplifies antimicrobial and anti-inflammatory effects and tissue repair. Notably, engineered probiotics loaded with berberine can be targeted to hypoxic regions within periodontal pockets, enabling antibiotic-free bacterial suppression and modulation of the inflammatory microenvironment (Li S. et al., 2025). Concurrently, mechanism-focused studies integrating network pharmacology and molecular docking are facilitating a shift from broadly pleiotropic activity toward more defined target engagement, providing a conceptual basis for the development of individualized treatment strategies.

Future research on berberine may focus on several priorities. First, continued optimization of delivery platforms (e.g., nanoparticles, hydrogels, and bioadhesive systems) is needed to further enhance solubility, bioavailability, and targeting, enabling site-specific formulations for distinct intraoral sites (e.g., periodontal pockets and ulcer surfaces). Second, the clinical evidence base should be expanded through multicenter, adequately powered trials with long-term follow-up to validate efficacy across diverse populations and disease severities, define optimal dosing and administration schedules, and establish standardized treatment protocols. Third, mechanistic investigations should be deepened by integrating multi-omics approaches to identify key targets and signaling networks through which berberine modulates oral diseases and to clarify its interactions with the oral microbiome and host immunity. Fourth, combination strategies require systematic evaluation, pairing berberine with conventional therapies (e.g., scaling and root planing or radiotherapy), other natural compounds, or emerging targeted agents to achieve synergy, particularly for oral cancer and refractory periodontitis. Finally, broader clinical applications should be explored, including potential roles in oral submucous fibrosis and radiation-induced oral mucositis, to facilitate translation of berberine from an adjunctive option toward a more central therapeutic modality and to support more precise and effective management of oral diseases.

## 10 Conclusion

In summary, berberine is a pharmacologically active compound derived from traditional medicine with established clinical use in several systemic conditions. Its antimicrobial, anti-inflammatory, antioxidant, bone-modulatory, and antitumor properties support its potential application in oral disease management, although disease-specific clinical efficacy in oral medicine remains to be validated through well-designed clinical studies. By continuously integrating emerging evidence and addressing current knowledge gaps, future research may further clarify the therapeutic value of berberine in oral diseases.

## Glossary

ADAMTS: A disintegrin and metalloproteinase with thrombospondin motifs
AIF: Apoptosis-inducing factor
AL: Attachment loss
ALP: Alkaline phosphatase
AMPK: AMP-activated protein kinase
BMSCs: Bone marrow–derived mesenchymal stem cells
BV/TV: Bone volume fraction
CAT: Catalase
CDK: Cyclin-dependent kinase
CHX: Chlorhexidine
CP: Camphorated phenol
DPSCs: Dental pulp stem cells
ECM: Extracellular matrix
EGFR: Epidermal growth factor receptor
EndoG: Endonuclease G
EPS: Extracellular polysaccharide
FADD: Fas-associated death domain protein
FAK: Focal adhesion kinase
FasL: Fas ligand
FC: Formocresol
gC1qR: Globular C1q receptor
GPR30: G protein–coupled estrogen receptor
GPx: Glutathione peroxidase
HDPFs: Human dental pulp fibroblasts
HGFs: Human gingival fibroblasts
HNSCC: Head and neck squamous cell carcinoma
hPDLSCs: Human periodontal ligament stem cells
hSCAPs: Human stem cells from the apical papilla
IκB: kinase β (IKKβ)
LOX: Lysyl oxidase
LPS: Lipopolysaccharide
LSCC: Laryngeal squamous cell carcinoma
MAO: Monoamine oxidase
MBC: Minimum bactericidal concentration
MCP-1: Monocyte chemoattractant protein-1
MIC: Minimum inhibitory concentration
MMP(s): Matrix metalloproteinase(s)
MtROS: Mitochondrial reactive oxygen species
NF-κB: Nuclear factor-κB
NOS: Nitric oxide synthase
NOX: NADPH oxidase
NQO1: NAD(P)H quinone oxidoreductase 1
NRF1/2: Nuclear factor erythroid 2–related factor 1/2
OSCC: Oral squamous cell carcinoma
OVX: Ovariectomized
PBE: *Phellodendron* bark extract
PC: Polyethyleneimine–cholesterol
PD: Probing depth
PDT: Photodynamic therapy
P-gp: P-glycoprotein
PI3K: Phosphoinositide 3-kinase
PLGA: Poly(lactic-co-glycolic acid)
PLP: Pyridoxal 5′-phosphate
PPARδ: Peroxisome proliferator–activated receptor delta
ROS: Reactive oxygen species
SIRT1: Sirtuin 1
SLNs: Solid lipid nanoparticsles
SOD: Superoxide dismutase
SrtA: Sortase A
T2DM: Type 2 diabetes mellitus
Tb.Th: Trabecular thickness
TC: Tetracycline
TRAIL: TNF-related apoptosis-inducing ligand
UCP2: Uncoupling protein 2
u-PA: Urokinase-type plasminogen activator
VEGF: Vascular endothelial growth factor
XO: Xanthine oxidase.
